# Depth specialization in mesophotic corals (*Leptoseris* spp.) and associated algal symbionts in Hawai'i

**DOI:** 10.1098/rsos.140351

**Published:** 2015-02-04

**Authors:** X. Pochon, Z. H. Forsman, H. L. Spalding, J. L. Padilla-Gamiño, C. M. Smith, R. D. Gates

**Affiliations:** 1 Environmental Technologies, Coastal and Freshwater Group, Cawthron Institute, Nelson, New Zealand; 2 Institute of Marine Science, University of Auckland, Auckland, New Zealand; 3 Hawai'i Institute of Marine Biology, University of Hawai'i, Kaneohe, HI, USA; 4 Department of Botany, University of Hawai'i at Mnoa, Honolulu, HI, USA; 5 Department of Biology, California State University Dominguez Hills, Carson, CA, USA

**Keywords:** mesophotic coral ecosystems, *Leptoseris*, *Symbiodinium* zooxanthellae, mitochondrial phylogenetics, depth specialization, coevolution

## Abstract

Corals at the lower limits of mesophotic habitats are likely to have unique photosynthetic adaptations that allow them to persist and dominate in these extreme low light ecosystems. We examined the host–symbiont relationships from the dominant coral genus *Leptoseris* in mesophotic environments from Hawai'i collected by submersibles across a depth gradient of 65–125 m. Coral and *Symbiodinium* genotypes were compared with three distinct molecular markers including coral (*COX1–1-rRNA* intron) and *Symbiodinium* (*COI*) mitochondrial markers and nuclear *ITS2*. The phylogenetic reconstruction clearly resolved five *Leptoseris* species, including one species (*Leptoseris hawaiiensis*) exclusively found in deeper habitats (115–125 m). The *Symbiodinium* mitochondrial marker resolved three unambiguous haplotypes in clade C, which were found at significantly different frequencies between host species and depths, with one haplotype exclusively found at the lower mesophotic extremes (95–125 m). These patterns of host–symbiont depth specialization indicate that there are limits to connectivity between upper and lower mesophotic zones, suggesting that niche specialization plays a critical role in host–symbiont evolution at mesophotic extremes.

## 2. Introduction

Light attenuation is a primary physical parameter that limits the distribution of coral reefs across depths and habitats [1]. In the tropics, photosynthetic corals are found at depths that range from *ca* 0 to 150 m in clear waters [1]. The stark differences in irradiance that occur over this depth gradient on spatial scales of only tens of metres have major implications for the distribution of coral species, and the genetic structure of populations [2,3]. The shallow and deep communities differ in species composition, reflecting physiological specialization and capacity tuned to specific corals, with depth being a proxy for the suite of parameters that change moving from shallow to deep communities. Disruptive selection along depth gradients has been proposed to lead to genetic divergence and possibly speciation despite the lack of obvious spatial barriers to gene flow [4–6]. In the case of scleractinian corals, coevolution of the host and symbiont is an important consideration for niche specialization and habitat partitioning, as there are trade-offs between different types of *Symbiodinium* dinoflagellates and host–symbiont specificities [7,8]. *Symbiodinium* spp. are likely to play a significant role in habitat partitioning and the ecological diversification of scleractinian corals along depth and habitat gradients. Striking patterns of depth-specific symbiont types have been reported in various coral species [9–12] and have been linked to differences in photo-physiological responses of different *Symbiodinium* types from shallow water (less than 14 m depth) dominant reef corals [13], or other depth-related environmental conditions acting synergistically such as temperature, salinity, pH, turbidity and nutrient availability [5,11].

Compared to shallow coral reef studies, mesophotic coral ecosystems have received very little attention because of logistical constraints and are just beginning to be explored [14–16]. The upper mesophotic (less than 60 m) is generally similar in community structure to shallow water ecosystems, whereas the lower mesophotic consists of a more distinct assemblage that is highly specialized to exceptionally low light conditions [14,16–19]. Vertical connectivity between shallow water and upper mesophotic zones is therefore of particular interest to understanding the resilience of shallow ecosystems to disturbance (i.e. the deep water refugia hypothesis; [2,5,20,21]). Deep reef ‘refugia’ areas are protected or dampened from disturbances that affect shallow reef areas and can provide a viable reproductive source for shallow reef areas following disturbance (reviewed in [5]). Mid-to-lower mesophotic zones, on the other hand, are ecologically very distinct, suggesting that connectivity would be limited across these zones, and that persistence in the lower mesophotic zone may require unique adaptations.

The genus *Symbiodinium* is phylogenetically diverse, consisting of nine divergent clades (A-I; [22]) and hundreds of different sub-clade types based on the internal transcribed spacer region 2 (*ITS2*) of nuclear ribosomal DNA [23,24], many of which arguably represent different species [25–27], but see 28. Despite numerous studies reporting striking patterns of host–symbiont specificity [29,30], biogeographic partitioning [31,32] and ecological zonation [2,11,13] of *Symbiodinium ITS2* types, the high variation among the copies of this gene found in individual genomes complicates interpretation and makes taxonomic assignment problematic [28,33–35]. Recent advances in genomic research [36–39] provide novel opportunities for the identification and characterization of alternative *Symbiodinium* markers, including a variety of nuclear, chloroplast and mitochondrial genes [40–42]. Previous studies characterizing *Symbiodinium* spp. diversity in mesophotic corals have all relied on the use of a single marker, *ITS2* [2,6,11,12,43–45]. Additional work is required to confront a wider range of available alternative markers and provide a more comprehensive understanding of the diversity and molecular taxonomy of *Symbiodinium* across contrasting environments.

**Figure 1.**
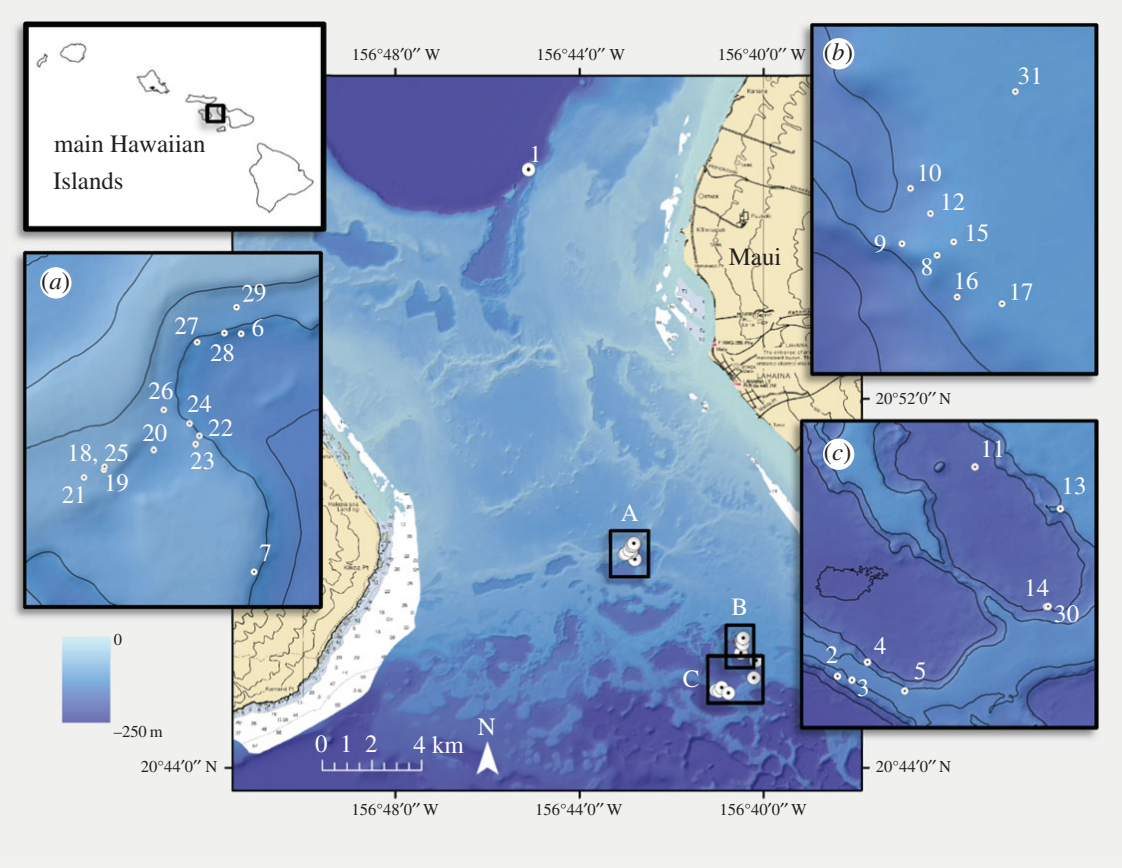
Map showing the 31 mesophotic sampling sites investigated in the ‘Au‘au Channel, Hawai'i.

The geographically isolated Hawaiian Archipelago is an excellent natural laboratory for studying speciation and adaptive radiation on land [46–48], and recent studies have shown similar patterns are present in the marine realm [49,50]. The genus *Leptoseris* is broadly distributed across depths within the Hawaiian Archipelago, presenting a unique experimental system to examine the potential for host–symbiont coevolution, speciation across a habitat gradient, and potential adaptive radiation across the Archipelago. Initial work on *Leptoseris* in Hawai'i reported the widespread presence of generalist *Symbiodinium* clades, and cryptic host diversity [43]; however, this general survey of host–symbiont diversity had limited sampling, and no attempt was made to taxonomically identify small fragments collected by submersible. More recent work has clarified the taxonomy of this genus by integrating molecular data with discrete microscopic features found in type specimens, showing close agreement between the coral genetic clades and skeletal microfeatures [51]. In addition, Luck *et al.* [51] found polyphyly between *Leptoseris* and *Pavona* and a putative new coral species, indicating that this group is in need of taxonomic revision. Luck *et al.* [51] also found trends suggesting possible depth zonation across the coral genetic clades; however, symbiont diversity was not examined. Here we sampled across the lower mesophotic depth gradient (between 65 and 125 m from the ‘Au‘au Channel; figure 1) in order to examine the genetic diversity of the coral genus *Leptoseris* and their associated symbiotic dinoflagellates using nuclear and mitochondrial markers.

## 3. Material and methods

### 3.1 Sample collection

Mesophotic corals (*n*=74) were collected across multiple depth gradients (65–125 m) using the Hawai'i Undersea Research Laboratory’s (HURL) manned submersibles, *Pisces IV* and *V*, during two cruises (January 2010 and February 2011) to the ‘Au‘au Channel (figure 1; see the electronic supplementary material, appendix A for sites coordinates) between the islands of Maui and Lāna'i aboard the R/V *Ka'imikai-o-Kanaloa*. In this study, we defined three mesophotic depth ranges: upper (65–75 m), mid (85–100 m) and lower (115–125 m). At each site along these depth ranges, representative corals approximately 20–30 cm in diameter were haphazardly selected from the middle of a *Leptoseris* reef, with each sample separated by at least 10 m in distance. A small, triangular piece of coral spanning from the middle to the outer edge of the coral head was removed using a Schilling Titan 4 manipulator arm, and placed in an individual sample container in the sampling basket. Collected samples were kept in a darkened container with ambient seawater and *in situ* temperatures, and processed in a darkened laboratory within 4 h of ascent to the surface. Each sample was photographed, sampled for DNA and then immediately frozen at −80°C.

### 3.2 DNA extraction, PCR and sequencing

Small biopsies of coral tissue (approx. 2 mm) were individually stored for a week in 600 μl of guanidium DNA extraction buffer [52]. All coral biopsies (*n*=74) were taken from the upper coenosarc region of coral fragments, and two additional biopsies (taken from the calyx and/or coenosarc region) were also taken from a subset of coral samples (*n*=12) haphazardly selected to cross the depth range of 75–125 m (electronic supplementary material, appendix A). These samples were used to determine whether different *Symbiodinium* mitochondrial *cytochrome c oxidase I* (*COI* mtDNA) genotypes would be found in different areas of the coral colony. Genomic DNAs from both the *Leptoseris* species and endosymbiotic *Symbiodinium* were co-extracted following [35].

Approximately 800 base pairs (bp) of a rapidly evolving intergenic spacer of *Leptoseris* spp. mitochondrial DNA (*cox1–1-rRNA* intron) was PCR-amplified using primers and thermocycling conditions described in [51]. PCR products were purified using the QIAquick PCR Purification Kit (Qiagen), and sequenced directly in both directions using the ABI Prism Big Dye Terminator Cycle Sequencing Ready Reaction Kit and an ABI 3100 Genetic Analyzer (Perkin-Elmer Applied Biosystems). All sequences were submitted to BLASTn search as well as compared to Luck *et al.* [51] sequence dataset for species-level identification.

A 1057 bp fragment of *Symbiodinium* spp. *COI* mtDNA was PCR-amplified using primers COX1_FOR2 and COX1_REV1, and the thermocycling conditions described in [41]. PCR products were purified and sequenced directly in both directions as described above. The *Symbiodinium* *ITS2* of nuclear ribosomal DNA (rDNA) marker was PCR amplified using protocols described in [22], and the primers ITS-DINO and ITS2REV2. The gene products were ligated into the pGEM-T Easy vector (Promega) and transformed into *α*-Select Gold Efficiency competent cells (Bioline). A minimum of 10 colonies were screened for inserts using plasmid-specific primers, and the positive screens were treated with exonuclease I and shrimp alkaline phosphatase and sequenced in both directions, as described above.

### 3.3 Phylogenetic analyses

DNA sequence chromatograms were inspected and bi-directional sequences were assembled using Sequencher v. 4.7 (Gene Codes Corporation, Ann Arbor, MI, USA), aligned with Clustal W implemented in BioEdit v. 5.0.9 [53] and manually refined. Three main DNA sequence alignments were generated (*Leptoseris* spp. *Cox1–1-rRNA* intron, *Symbiodinium* *COI* mtDNA and *Symbiodinium* *ITS2* rDNA). Additionally, a fourth comparative sequence alignment of *cox1–1-rRNA* intron was created, including all Luck *et al.* [51] sequences and one representative sequence from each clade reported in this study. The *Leptoseris* spp. *Cox1–1-rRNA* phylogenies were rooted using *Siderastrea radians* from whole mitochondrial genomes available in GenBank (DQ643838). The *Symbiodinium* *COI* phylogeny was rooted using *Symbiodinium* *F*_1_ described in [41] (GenBank JN558066). Both genes were analysed independently using maximum-likelihood (ML) and Bayesian methods. Best-fit models of evolution and ML inferences with global tree searching procedure (10 starting trees) were estimated using Treefinder v. 12.2.0 [54]. Robustness of phylogenetic inferences was estimated using the bootstrap method [55] with 1000 pseudoreplicates in all analyses. Bayesian analyses were performed using the parallel version of MrBayes v. 3.1.2 [56,57], starting from a random tree of four chains with two runs of Metropolis-coupled Markov chain Monte Carlo, and including 1 000 000 generations with sampling every 10 generations. The average standard deviation of split frequencies was used to assess the convergence of the two runs. In all cases, the chains converged within 0.25 generations. Therefore, the first 25 000 trees were discarded as burn-in and a 50% majority-rule consensus tree was calculated from the remaining 75 000 trees. Nodal support was reported as Bayesian posterior probabilities.

*Symbiodinium* *ITS2* cloned sequences were identified by local BLASTn search against the clade C alignment available in the GeoSymbio database [24], as well as BLASTn search against NCBI. To avoid overestimating *Symbiodinium* diversity owing to the high intragenomic variability of the *ITS2* gene [34,35], sequences included in the downstream analyses followed the same conservative criteria as used in our previous studies [8,43,58]. Statistical parsimony haplotype networks of *Symbiodinium* *ITS2* rDNA sequences and *Symbiodinium COI* sequences were constructed using the software TCS v. 1.21 [59] with a 95% connection limit and gaps were treated as a fifth state.

### 3.4 Statistical analyses

Patterns of host–symbiont association across collection sites and depth gradients were tested statistically using the square-root of the relative frequency of *Symbiodinium COI* sequence genotypes present in each *Leptoseris* spp. sample using the Bray–Curtis coefficient of similarity (*S*) in the software package Primer v. 6 [60]. To test for the partitioning of *Symbiodinium* genotypes by host (i.e. *Symbiodinium* versus *Leptoseris* mtDNA genotypes), collection site (i.e. between the 31 collection sites), and collection depth (i.e. between depth ranges 65, 75, 85, 95, 100, 115 and 125 m), a permutational MANOVA [61–63] was performed with ‘host’, ‘site’ and ‘depth’ as fixed factors. The test was performed using Type 1 sums of squares and unrestricted permutation of raw data. Because the *Symbiodinium* *ITS2* sequences were obtained from a relatively limited subset of coral samples (*n*=14 out of the 77 samples investigated), an independent permutational MANOVA analysis was performed to test for the partitioning of *Symbiodinium* *ITS2* sequences by symbiont and host mtDNA genotypes and by depth only.

## 4. Results

### 4.1 Phylogenetic analyses

**Figure 2.**
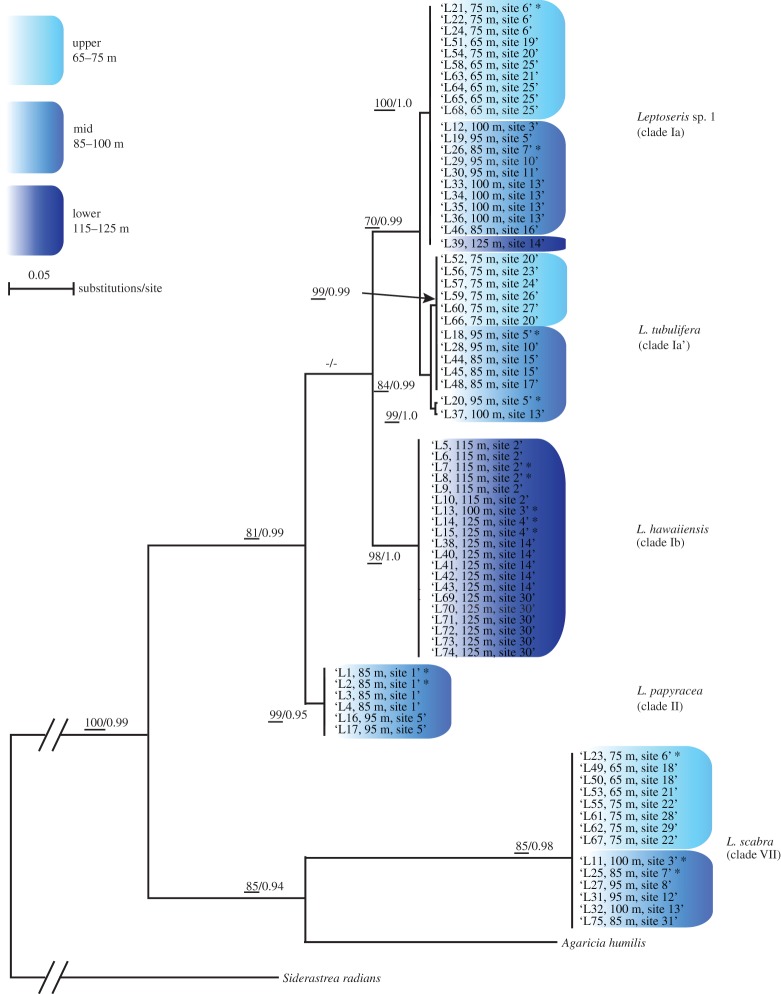
Best ML topology for *Leptoseris* spp. based on 74 mitochondrial *COX1–1-rRNA* intron sequences (alignment size: 818 bp). Numbers at nodes represent the ML bootstrap support values greater than 70% (underlined numbers) and Bayesian posterior probabilities greater than 0.8. Dashes (–) indicate statistically unsupported nodes. The phylogram was rooted using the coral *Siderastrea radians*. Collection depth ranges of coral samples are highlighted in blue for upper, mid and lower mesophotic (see inside legend). Tip names correspond to the sample IDs (letter L followed by a number), binned collection depth, and the collection site number. All samples (*n*=74) were genotypes using the *COI* gene (figures 3–5), and a subset (*n*=14; see asterisks (*) sign following tip names) were genotyped using *ITS2*. A detailed list of collection depths and dates, as well as sampling sites with latitude/longitude coordinates, the coral cover at each site, number of *ITS2* sequence variants per sample, and all *COX-1–1-rRNA* GenBank accession numbers is provided in the electronic supplementary material, appendix A.

High-quality sequences of *COX1–1-rRNA* mtDNA were obtained for all investigated *Leptoseris* spp. samples (*n*=74). The sequence alignment was 818 bp in length. The model of evolution calculated in Treefinder v. 12.2.0 corresponded to the *GTR*+*G*+*I* model [64]. All Bayesian analyses yielded similar ‘burn-in’ curves. Standard deviation of split frequencies were well below 0.01 after *ca* 15 000 generations, and the Potential Scale Reduction Factor reached the value of 1 for all parameters. Phylogenetic reconstructions recovered five divergent and highly supported clades, each corresponding to known *Leptoseris* species previously described in [51] (figure 2). Additional phylogenetic analysis, including all sequences from Luck *et al.* [51] and a representative sequence from each clade reported here, indicated unambiguous correspondence for *Leptoseris* sp. 1 (clade Ia), *Leptoseris tubulifera* (here referred to as clade Ia’), *Leptoseris hawaiiensis* (clade Ib) and *Leptoseris scabra* (clade VII) (electronic supplementary material, appendix B). The remaining clade (clade II) was most similar by genetic distance measures to *Leptoseris papyracea* but sequences differed by up to 21 bp. *Leptoseris scabra* was the most divergent with respect to other *Leptoseris* species, and consistent with Luck *et al.*’s [51] finding that *L. scabra* was polyphyletic with *Pavona* and *Agaricia*; this species may require future generic reassignment.

*Leptoseris scabra* (clade VII) was exclusively represented by samples collected at upper and mid mesophotic zones, with approximately the same number of samples collected from 65 to 75 m (*n*=8) and from 85 to 100 m (*n*=6) depth ranges, respectively (figure 2). Among the more closely related *Leptoseris* species, *L. tubulifera* (clades Ia’) and *Leptoseris* sp. 1 (clade Ia) were found from upper and mid mesophotic similarly to *L. scabra*. *Leptoseris* sp. 1 was also detected once (sample no. L39) from the lower (115–125 m) depth range. *Leptoseris papyracea* (clade II) and *L. hawaiiensis* (clade Ib) were exclusively found at mid and deep water (115–125 m) depth ranges, respectively (figure 2). In Luck *et al.* [51], the water-depth ranges for these five species were 70–80 m (*Leptoseris* sp. 1), 20–85 m (*L. tubulifera*), 80–130 m (*L. hawaiiensis*), 40–70 m (*L. papyracea*) and 70–130 m (*L. scabra*).

**Figure 3.**
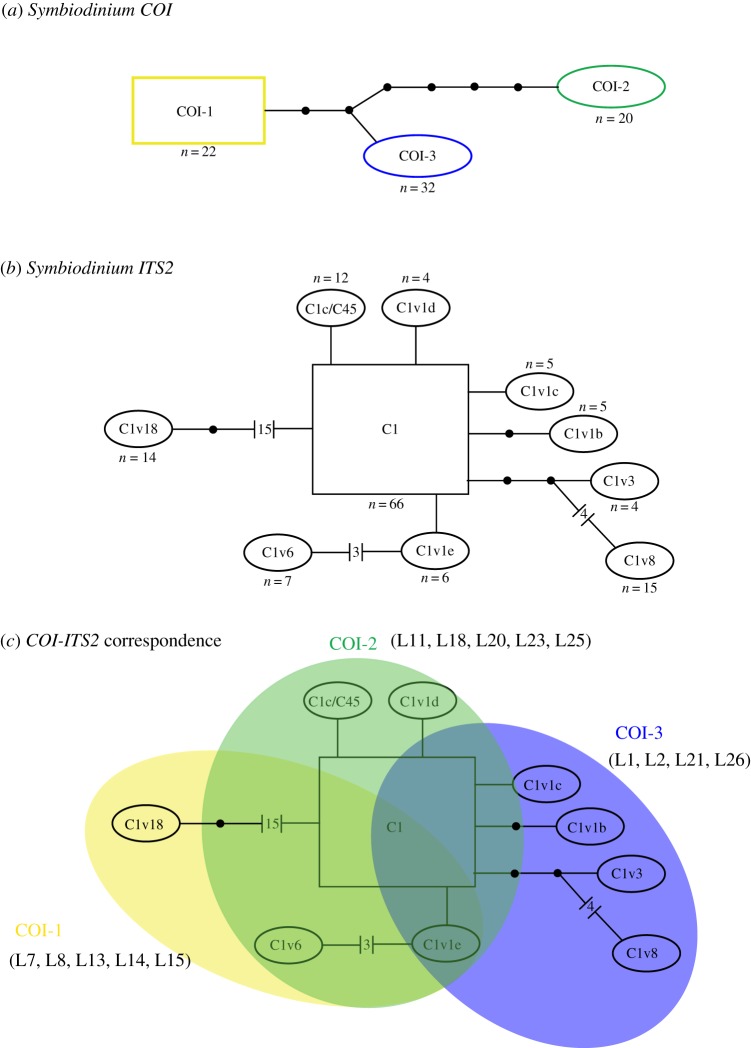
Genotype networks obtained by statistical parsimony in the program TCS v1.21, showing the relationships between sequence haplotypes for (*a*) the *Symbiodinium* *COI* gene, (*b*) the *Symbiodinium* *ITS2* gene and (*c*) an overlap schematics of the correspondence between *Symbiodinium* *COI* and *ITS2* sequence haplotypes (samples selected for *ITS2* sequence typing are shown in parentheses; see also figure 2). Each line in the network represents a single base-pair change. The black dots between some lines represent hypothetical intermediate mutations. The root for each network (estimated by the algorithm) is represented as a rectangle. A summary of *Leptoseris* spp. samples and associated *Symbiodinium* *COI* and *ITS2* sequences can be found in the electronic supplementary material, appendix A.

High-quality sequences of *Symbiodinium* *COI* mtDNA sequences were obtained for all investigated *Leptoseris* spp. samples (*n*=74). Sequence alignment was 1057 bp in length. All *COI* sequences belonged to *Symbiodinium* clade C and were different from the previously published *COI* sequences produced in [41] for *Symbiodinium* C1 (4–6 bp differences), C15 (3–7 bp), C90 (13–14 bp) and C91 (14–17 bp) (data not shown). The model of evolution calculated in Treefinder v. 12.2.0 corresponded to the HKY model [65]. Phylogenetic reconstructions yielded three distinct and well-supported *COI* sequence haplotypes, with haplotypes COI-1 (*n*=22) and COI-3 (*n*=32) more closely related to one another than COI-2 (*n*=20) (electronic supplementary material, appendix C). The relationship and number of bp differences between the three *Symbiodinium* *COI* haplotypes can be visualized in the statistical parsimony network of figure 3*a*. The *COI* haplotypes differed by between 3 and 7 bp. Identical *COI* *Symbiodinium* haplotypes were recovered from all 12 *Leptoseris* spp. samples that were subjected to additional *COI* genotyping from calyx and/or coenosarc coral biopsies (see the electronic supplementary material, appendix A).

A subset (*n*=14) of samples representing all three *Symbiodinium* *COI* genotypes and the five *Leptoseris* species (figure 2; electronic supplementary material, appendix A) was selected for cloning and sequencing of the *Symbiodinium* spp. *ITS2* gene. A total of 140 *ITS2* sequences were obtained, including between 8 and 12 cloned sequences per sample (average of 10 sequences per sample; see the electronic supplementary material, appendix A). Ten *Symbiodinium* spp. *ITS2* genotypes were recovered, including three previously published types (C1, C1c/C45 and C1v1b), and seven novel sequence variants (C1v1c, C1v1d, C1v1e, C1v3, C1v6, C1v8 and C1v18) that differed from *Symbiodinium* type C1 by 1–18 bp (figure 3*b*). These novel sequences were named ‘C1v’ followed by an alphanumeric descriptor following the naming system of Chan *et al*. [43]. Between two and six co-occurring *ITS2* sequence types were recovered from individual coral samples, with type C1 common in all samples (electronic supplementary material, appendix A). The four most common *Symbiodinium* *ITS2* sequence types were C1 (*n*=66), C1v8 (*n*=15), C1v18 (*n*=14) and C1c/C45 (*n*=12).

Patterns of correspondence were observed between the *Symbiodinium* spp. *COI* haplotypes and specific *ITS2* community sequence profiles (figure 3*c*). While *ITS2* type C1 and C1v1e were shared by at least one coral sample harbouring one of the three *COI* haplotypes, several other *ITS2* sequence types were restricted to a specific *COI* haplotype. For example, *ITS2* sequence type C1v18 was uniquely associated with haplotype COI-1, *ITS2* sequence types C1v1d and C1c/C45 were restricted to haplotype COI-2, and *ITS2* sequence types C1v1b, C1v1c, C1v3 and C1v8 were restricted to haplotype COI-3 (figure 3*c*; electronic supplementary material, appendix A).

All novel DNA sequences were submitted to GenBank. *Symbiodinium* spp. *COI* mtDNA sequences can be found under accession numbers HG942426 (COI-1), HG942427 (COI-2) and HG942428 (COI-3). *Symbiodinium* spp. *ITS2* rDNA sequences can be found under AF333515 (C1, [23]), EU449103 (C1c/C45, [23]), FJ919244 (C1v1b, [43]), HG942429 (C1v1c), HG942430 (C1v1d), HG942431 (C1v1e), HG942432 (C1v3), HG942433 (C1v6), HG942434 (C1v8) and HG942435 (C1v18). All *Leptoseris* spp. *COX1–1-rRNA* mtDNA sequences have been deposited under HG942436–HG942509 (see the electronic supplementary material, appendix A for more details).

### 4.2 Host–symbiont partitioning of mitochondrial genotypes

**Figure 4.**
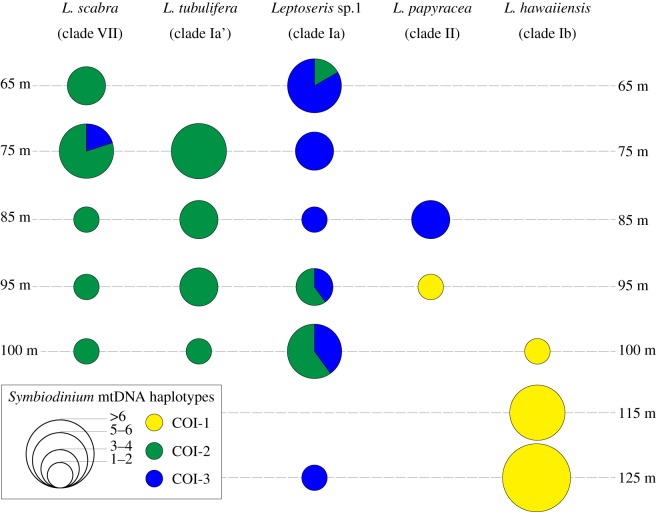
Partitions of *Symbiodinium* spp. *COI* haplotypes by host species and by collection depth. Proportions of *COI* haplotypes in each *Leptoseris* species and for each collection depth are indicated by the pie charts. Sizes of pie charts are proportional to the number of samples investigated (see circular inset scale).

Comparison of *Symbiodinium* spp. and *Leptoseris* spp. mtDNA datasets indicated genetic partitioning between host–symbiont genotypes and between habitats. Figure 4 shows the partitioning of *Symbiodinium* spp. *COI* haplotypes by host species/clades and by collection depth. *Leptoseris scabra* (clade VII) associated almost exclusively with *Symbiodinium* COI-2 (*n*=13) and only one sample associated with COI-3. All *L. tubulifera* (clade Ia’) samples (*n*=13) associated with COI-2. *Leptoseris* sp. 1 (clade Ia) samples associated primarily with *Symbiodinium* COI-3 (*n*=15), and less frequently with COI-2 (*n*=6). *Leptoseris* *papyracea* (clade II), samples collected at 85 m depth all harboured exclusively COI-3 (*n*=4), whereas the remaining two samples that were collected at 95 m depth associated with *Symbiodinium* COI-1. Finally, the deep water coral *L. hawaiiensis* (clade Ib) (*n*=20) associated exclusively with COI-1.

### 4.3 Statistical analyses

To test the observed partitioning of *Symbiodinium* spp. *COI* mtDNA haplotypes between host mtDNA genotypes, collection sites and mesophotic depth ranges (figure 4; electronic supplementary material, appendix A), a permutational MANOVA was performed (table 1*a*). *Symbiodinium* *COI* haplotypes were significantly different between host genotypes, sites and depth, and there was a significant *host*×*site* interaction (i.e. coral mtDNA genotypes associated with different *Symbiodinium* mtDNA haplotypes at each site), as well as a significant *host*×*depth* interaction (i.e. coral mtDNA genotypes associated with different *Symbiodinium* mtDNA haplotypes at each mesophotic depth ranges). Owing to limitations in the number of individual coral colonies that were collected at each site and between depth, calculations of *depth*×*site* interaction and *host*×*depth*×*site* interaction were not compared.

**Table 1.** Permutational MANOVA for (*a*) *Symbiodinium* spp. *COI* haplotypes by host genotype, collection depth and sites, and (*b*) *Symbiodinium* spp. *ITS2* sequence profiles by symbiont and host mtDNA haplotype, and collection depth. (Significant *p*-values are indicated with an asterisk, *p<0.05.)

source	d.f.	pseudo-F	*p*-value
(*a*)			
host (five host species)	6	71.469	0.001*
depth (seven water-depth ranges)	4	82.667	0.001*
site (31 sites)	24	6.5357	0.001*
*host*×*depth*	6	4.126	0.001*
*host*×*site*	2	12.188	0.001*
(*b*)			
symbiont (three *Symbiodinium* mtDNA genotypes)	2	14.782	0.001*
host (five *Leptoseris* mtDNA genotypes)	2	0.7877	0.579
depth (six water-depth ranges)	4	1.4863	0.188
*symbiont*×*depth*	1	0.12057	0.964

To test whether the *Symbiodinium* spp. *ITS2* sequence profiles observed in individual coral colonies (figure 3; electronic supplementary material, appendix A) partitioned in a similar manner as the *COI* gene, an additional permutational MANOVA was performed (table 1*b*). *Symbiodinium* *ITS2* sequence profiles recorded in each of 14 *Leptoseris* spp. colonies correlated significantly with the *Symbiodinium* *COI* haplotypes (*p*=0.001*), but were not significantly different between host genotypes (*p*=0.579) or depth (*p*=0.188).

## 5. Discussion

This study investigated the genetic patterns in *Leptoseris* spp., the dominant reef-building coral genus in mesophotic ecosystems in the Hawaiian Archipelago, and its associated *Symbiodinium* dinoflagellates. Owing to the difficulties of conducting research in the mesophotic zone [16,44,66], previous studies of coral–algal associations have been largely limited to upper mesophotic environments (i.e. 30–60 m depth [2,6,11,12,44]). Using a combination of nuclear and mitochondrial markers, we reveal highly specific host–symbiont associations, and strong evidence for depth-related niche partitioning of these associations particularly between *L. hawaiiensis* and congeners collected from Hawai'i over a 65–125 m depth range. This study brings new insights into the molecular diversity and adaptation of *Leptoseris*–*Symbiodinium* associations in extreme light-limiting environments.

### 5.1 Mitochondrial markers resolve *Leptoseris–Symbiodinium* associations

Most mitochondrial DNA regions are notorious for slow evolution and lack resolution for distinguishing between congeneric anthozoans [67,68]. Luck *et al.* [51] recently conducted comprehensive morpho-molecular analyses to reveal that the *cox1–1-rRNA* intron was informative across several Agariciid genera. Here, we surveyed five species of *Leptoseris*, four of which (*Leptoseris* sp. 1, *L. tubulifera*, *L. hawaiiensis* and *L. scabra*) unambiguously corresponded to species described in [51] while the remaining clade differed from *L. papyracea* by 21 bp (electronic supplementary material, appendix B). Additional analysis of skeletal micromorphological ornamentation is required to determine whether the latter specimens correspond to *L. papyracea* or represent a different species. Nevertheless, this new investigation confirms that the *cox1–1-rRNA* intron is an informative genetic marker for *Leptoseris* spp. and may foster valuable comparative studies between mesophotic coral communities in Hawai'i as well as in higher diversity regions such as the Indo-West Pacific.

Our knowledge of *Symbiodinium* evolution has historically been constrained by the limited number of phylogenetic markers that have been applied to this group, with nuclear and chloroplast ribosomal genes largely dominating phylogenetic investigations (reviewed in [28]). The *ITS2* is by far the most common marker used to decipher fine-scale patterns within the nine existing *Symbiodinium* clades [23,69–71] and previous studies investigating host–symbiont diversity and specificity along mesophotic gradients have all relied on this marker [2,6,11,12,43–45]. However, frequent intragenomic variation between ITS copies within an individual *Symbiodinium* genome (as approximated by clonal culture cell lines; [34]) makes taxonomic assignments problematic [28]. Consequently, the topic of interpreting ecological patterns of *Symbiodinium* using *ITS2* has generated intense debate and significant emphasis has been placed on methodological limitations rather than on the complex nature of the marker itself [33–35,72,73]. We recovered three *ITS2* types (C1, C1c, C1v1b) identical to [43]; however, our dataset also revealed seven novel *ITS2* sequence variants (C1v1c, C1v1d, C1v1e, C1v3, C1v6, C1v8 and C1v18).

Mitochondrial Cytochrome Oxidase I (*COI*) is an important enzyme in aerobic metabolism in prokaryotes and eukaryotes [74] and is best known as the molecule used in barcoding a diversity of animals and other eukaryotes [75], including *Symbiodinium* [76]. In contrast with the *ITS2* data, three clearly distinct *Symbiodinium* haplotypes were recovered in *Leptoseris* spp. using the *COI* marker. Notably, *COI* haplotypes were obtained via direct sanger sequencing, limiting the possibility of incorporating biases owing to methodological artefacts, such as chimaeras formation, through cloning and sequencing [34,73]. The three unambiguous *COI* haplotypes yielded an appreciable level of resolution (i.e. 3–7 bp differences) considering their close association with related genotypes within *Symbiodinium* clade C and in contrast with other studies showing very limited resolution between distinct *Symbiodinium* clades using this marker [42,77]. Our results confirm a previous observation [41] that the *COI* marker displays unexpectedly high levels of sequence divergence between some symbiont types within clade C, possibly linked to the mode of symbiont transmission and/or reflecting different selection pressures from unusual environments. For example, the *COI* resolution is minimal (1 bp change) between common shallow water generalist symbiont types C1 and C3 (X. Pochon 2015, unpublished data) but yielded evolutionary rates similar to *ITS2* between the vertically transmitted foraminifera-specific symbiont types C90 and C91 [41]. Similarly, three scenarios might explain the higher resolution of *Leptoseris* symbionts using *COI*: (i) faster lineage sorting by the mitochondrial locus, (ii) slowed concerted evolution or paralogous copies of the *ITS2* marker, and/or (iii) the mitochondrial marker is under selection pressures owing to the extreme habitat conditions. Finally, the high-quality sequences obtained from all investigated *Leptoseris* spp., including 12 samples that were subjected to additional *COI* genotyping from calyx and/or coenosarc coral biopsies, indicated the presence of a single *COI* haplotype per colony. This result suggests the presence of a single symbiont type per *Leptoseris* specimen, corroborating previous high-resolution markers studies indicating that *in hospite* populations of *Symbiodinium* are often, but not always [8,78], comprised one highly clonal *Symbiodinium* genotype [79–81]. The ease of directly sequencing and aligning the *Symbiodinium* mitochondrial marker thus provide opportunities for future work on symbiosis ecology in Agaricidae and other corals.

### 5.2 Depth specialization and coevolution of *Leptoseris* species and associated *Symbiodinium*

This study echoes the findings of several studies that have found marked zonation by depth in scleractinian corals [4–6,82]. We revealed patterns of depth zonation in both *Leptoseris* coral and associated *Symbiodinium*, particularly with regards to *L. hawaiiensis*. Similar to [51], both *Leptoseris* sp. 1 (Clade Ia) and *L. tubulifera* (clades Ia’) were distributed from upper (more than 65 m) to mid (less than 100 m) mesophotic ranges, *L. papyracea* was confined to mid-range, and *L. hawaiiensis* was restricted to deeper (more than 100 m) environments. The frequencies of *Symbiodinium COI* clades differed significantly by depth, and by host clade. The host clade *L. hawaiiensis* (clade Ib) was found exclusively at the deepest mesophotic depths, and it only harboured one genotype of *Symbiodinium:* haplotype COI-1 (figures 2 and 4). Haplotype COI-1 was only found at or below 95 m, indicating this clade is likely to be uniquely adapted to the low light conditions of the lower mesophotic zone. Mesophotic reefs in the ‘Au‘au Channel occur between 30 and 150 m [18], and light dramatically attenuates with only around 1% of surface light penetrating beyond the threshold of 97 m, and only 0.1% penetrating to the lower extremes of 150 m [83,84]. These light values fall well below the minimal light levels (more than 50 μE m^−2^ s^−1^) that are thought to define the lower limits (usually *ca* 40–50 m depth) of coral reef development [1], yet these corals persist and dominate at these extremes most likely owing to specialized adaptations from both the host and symbiont [85].

**Figure 5.**
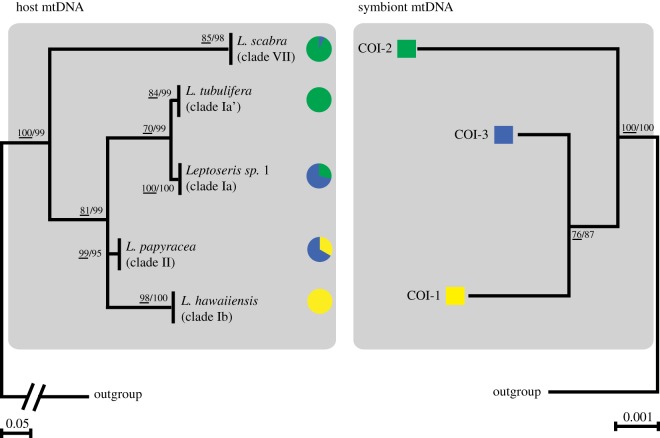
Links between *Leptoseris* species and *Symbiodinium* mtDNA genotypes represented as mirrored host–symbiont phylogenies. Phylograms correspond to the best ML topology for *Leptoseris* species (*a*) and *Symbiodinium* (*b*) mtDNA sequence haplotypes. Numbers at nodes represent the ML bootstrap support values (underlined) and Bayesian posterior probabilities (in percentage). Unsupported nodes (less than 50%) were manually collapsed. Coloured pie charts represent the frequencies of *Symbiodinium* mtDNA haplotypes found in each *Leptoseris* species.

Coadaptation and coevolution appear to be consistent with both host and symbiont phylogenies, and dominance in the deepest mesophotic zone is more recently derived in both phylogenetic trees. Figure 5 represents putative evolutionary links between *Leptoseris* spp. and *Symbiodinium* spp. mtDNA genotypes. Clear host–symbiont associational patterns were observed when highlighting the most common mtDNA associations such as *L. scabra* and *L. tubulifera* with *Symbiodinium* spp. COI-2, *Leptoseris* sp. 1 and *L. papyracea* with *Symbiodinium* spp. COI-3, and *L. hawaiiensis* with *Symbiodinium* spp. COI-1. Interestingly, *L. scabra* (clade VII) and *Symbiodinium* spp. COI-2 were resolved as most divergent in their respective phylogenies (figure 5), and COI-2 was the only *Symbiodinium* haplotype that was not recovered in sites deeper than 100 m depth (figure 4). Similar coral patterns were found by Luck *et al.* [51], with depth restriction occurring in more derived positions of the phylogenetic tree for relatively few coral clades (*L. hawaiiensis* and *L. scabra*). The distribution of symbionts across depths and host lineages (figure 4) is consistent with niche partitioning and expansion of the host range into sub-optimal low light habitats, although this hypothesis remains to be rigorously tested. Similarly, the significant *host*×*site* interaction uncovered in this study (table 1) suggests potential geographical or habitat patterns in the ecological distribution of mesophotic *Leptoseris*. However, further work and additional sampling is required to test these hypotheses.

Deep reefs have been proposed as potential refugia for shallow reef organisms and as such, connectivity across depth gradients is of particular interest. The general focus of interest for deep water refugia is the vertical connectivity between shallow reefs (30–60 m) and the upper mesophotic zone (less than 60 m) [19]. This study focused on the lower mesophotic zone (65–125 m), sampling across a known transition zone in benthic community structure at approximately 100 m depth [16,19]. Although reduced connectivity across these depths might be expected, the finding of host–symbiont coevolution and depth zonation indicate unique adaptations and niche specialization, with strongly limited genetic connectivity between depths.

Pronounced evolutionary divergence across depth has been discovered in several coral species in the Caribbean across both the host and symbiont [12,82]. Similarly, patterns of within species population genetic structure have indicated strong signals of segregation by depth for host genotypes and symbiont types [4–6]. This study focused only on the lower and extreme mesophotic depths (65–125 m), providing, to our knowledge, the first evidence for symbiont specialization deeper than 100 m, and confirming general host zonation patterns suggested by Luck *et al.* [51]. This study lays the foundation for future work to investigate: (i) the possibility of recent adaptation and radiation into extreme depths (requiring broader taxonomic sampling for both host and symbiont to observe multiple occurrences of similar patterns), (ii) the direction of genetic migration (e.g. from asexual fragments rolling downward) and the mechanism for speciation by depth (e.g. competitive exclusion to deeper marginal habitats), (iii) the role of geographical isolation and host–symbiont depth specialization, and (iv) the particular genetic loci that may be linked to physiological requirements involved in deep water adaptation. Despite the technical challenges associated with extreme depths, mesophotic corals are likely to hold important clues to understanding niche specialization and adaptation.

## Supplementary Material

Table S1: Detailed Summary Table. Detailed list of collection depths and dates, as well as sampling sites with latitude/longitude coordinates and the coral cover observed around each of the 74 Leptoseris spp. samples investigated in this study. Figure S1: Phylogenetic reconstruction of the coral genus Leptoseris. Best Maximum likelihood (ML) topology for Leptoseris spp. based on 79 mitochondrial COX1-1-rRNA intron sequences (alignment size: 751 bp), including 73 sequences from Luck et al. (2013) and 6 representative sequences from this study. Figure S2: Phylogenetic reconstruction of Symbiodinium symbionts. Best Maximum likelihood (ML) topology for Symbiodinium spp. based on 74 mitochondrial COI sequences (alignment size: 1057 bp).

## Data accessibility

Appendix A is available from the Dryad Digital Repository: http://doi.org/10.5061/dryad.jt8hj. Sequence data have been deposited under GenBank accession nos. HG942426–HG942428 and HG942436– HG942509.
